# New-onset refractory status epilepticus due to autoimmune encephalitis after vaccination against SARS-CoV-2: First case report

**DOI:** 10.3389/fneur.2022.946644

**Published:** 2022-08-16

**Authors:** Jana Werner, Giovanna Brandi, Ilijas Jelcic, Marian Galovic

**Affiliations:** ^1^Department of Intensive Care Medicine, University Hospital Zurich, Zurich, Switzerland; ^2^Department of Neurology, Clinical Neuroscience Center, University Hospital Zurich, Zurich, Switzerland

**Keywords:** new-onset refractory epileptic state, NORSE, SARS-CoV-2 vaccination, BNT162b2, seronegative autoimmune encephalitis, postvaccinal encephalitis

## Abstract

**Background:**

Vaccination against SARS-CoV-2 has been conducted frequently to limit the pandemic but may rarely be associated with postvaccinal autoimmune reactions or disorders.

**Case presentation:**

We present a 35-year-old woman who developed fever, skin rash, and headache 2 days after the second SARS-CoV-2 vaccination with BNT162b2 (Pfizer/Biontech). Eight days later, she developed behavioral changes and severe recurrent seizures that led to sedation and intubation. Cerebral magnetic resonance imaging showed swelling in the (para-) hippocampal region predominantly on the left hemisphere and bilateral subcortical subinsular FLAIR hyperintensities. Cerebrospinal fluid analysis revealed a lymphocytic pleocytosis of 7 cells/μl and normal protein and immunoglobulin parameters. Common causes of encephalitis or encephalopathy such as viral infections, autoimmune encephalitis with well-characterized autoantibodies, paraneoplastic diseases, and intoxications were ruled out. We made a diagnosis of new-onset refractory status epilepticus (NORSE) due to seronegative autoimmune encephalitis. The neurological deficits improved after combined antiepileptic therapy and immunomodulatory treatment including high-dose methylprednisolone and plasma exchange.

**Conclusions:**

Although a causal relationship cannot be established, the onset of symptoms shortly after receiving the SARS-CoV-2 vaccine suggests a potential association between the vaccination and NORSE due to antibody-negative autoimmune encephalitis. After ruling out other etiologies, early immunomodulatory treatment may be considered in such cases.

## Introduction

Adverse effects of SARS-CoV-2 vaccination with mRNA vaccines are rare and mostly limited to local reactions or slight systemic symptoms (1) including fatigue and headache most commonly. Generally, post-vaccination autoimmune encephalitis is rare (2, 3), but it has been reported to have an association with the adenoviral vector vaccine ChAdOx1 (Oxford-AstraZeneca) (4) as well as the mRNA vaccine (Pfizer/Biontech) for SARS-CoV-2 (3, 5).

A heterogeneous group of sporadic autoimmune encephalitis cases associated with SARS-CoV-2 infection has also been reported (6). Additionally, more and more autoimmune encephalitis cases have been linked to other viral triggers (7).

Here, we describe a case of new-onset refractory status epilepticus (NORSE) due to autoantibody-negative autoimmune encephalitis in close temporal association with the second SARS-CoV-2 vaccination dose of BNT162b2 (Pfizer/Biontech).

## Case presentation

A 35-year old woman was referred to our center with recurrent focal to bilateral convulsive seizures after an episode of fever, headache, and skin rash 6 days prior. Two days before the onset of fever, she received the second dose of an mRNA-based SARS-CoV-2 vaccine (BNT162b2, Pfizer/Biontech). The first dose was well-tolerated. The patient's medical history revealed only sporadic but not recent cocaine consumption and mild SARS-CoV-2 infection a year before the vaccination.

**Figure 1:**
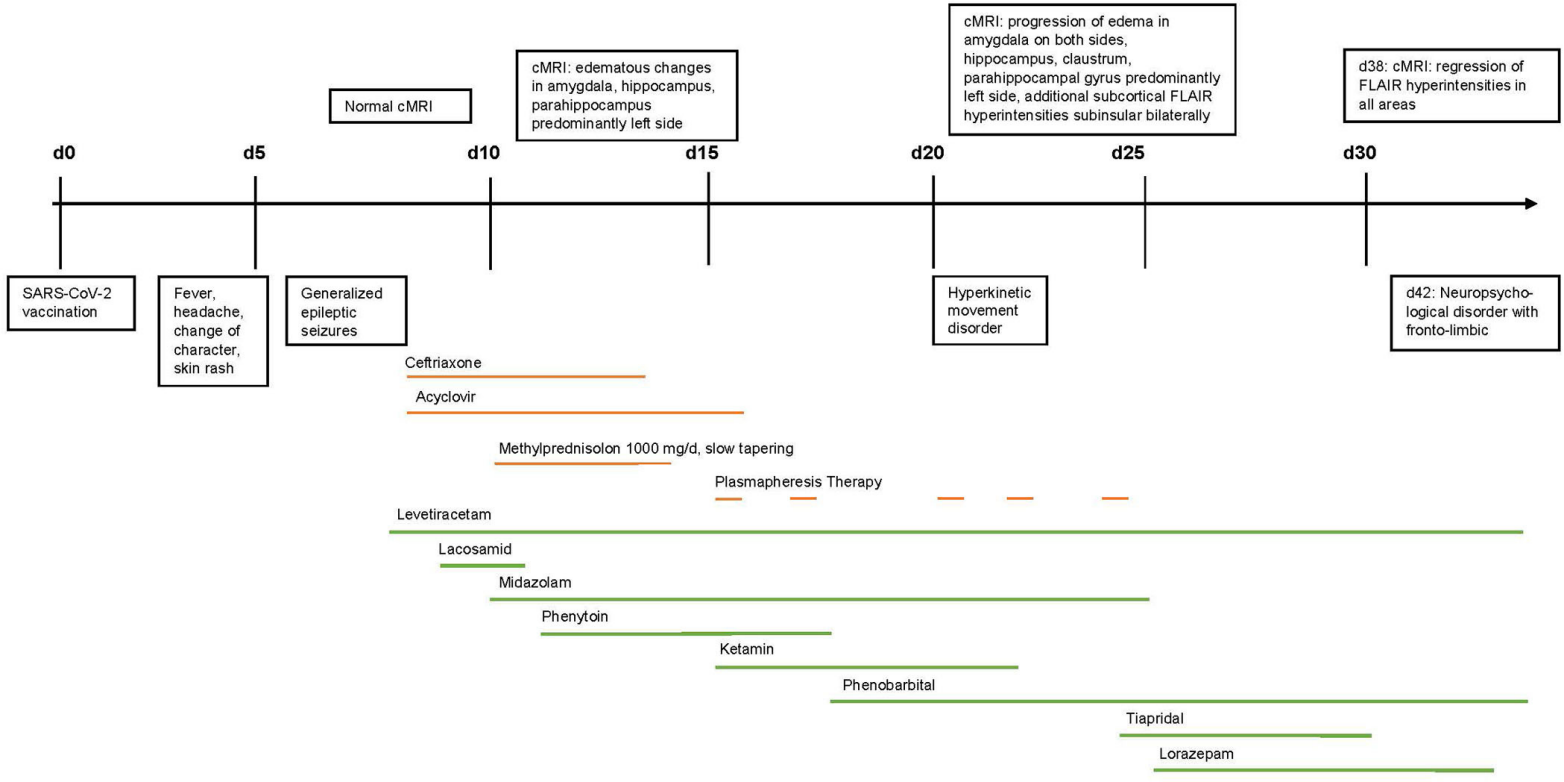
Timeline of clinical development, therapy, and MRI-findings.

The clinical, laboratory, and imaging findings are summarized in Table 1 and are illustrated in Figure 1. On admission, the patient presented with fever of up to 40°C, visual impairment, behavioral changes, recurrent focal to bilateral tonic-clonic seizures, reduced level of consciousness, and choreatic movements. On admission, moderately elevated TSH, normal free T3 and T4 hormone levels, and slightly elevated liver function tests possibly due to recent overuse of paracetamol were found in the blood test. Drug screening of urine was negative.

**Table 1:** **(A)** Symptoms and **(B)** therapy, **(C)** imaging, **(D)** laboratory, **(E)** CSF, and **(F)** EEG findings.

**(A) Symptoms**		
SARS-CoV-2 vaccination	Day 0	
Fever, headache, change of character, skin rash	Day 2–7	
Generalized epileptic seizures	Day 7	
Hyperkinetic movement disorder	Day 22	
APE^2^ Score	10	
Neuropsychological findings	Day 42: severe neuropsychological disorder with fronto-limbic dysfunction	
**(B) Therapy**		
Therapy with acyclovir 3 × 10 mg/kg body weight/d iv	Day 8–16, until negative PCR of VZV and HSV	
Therapy with ceftriaxone 2 × 2,000 mg/d	Day 8–14	
Cotrimoxazol 800/160 mg	Three times a week during high doses steroid treatment	
Therapy with methylprednisolone 1,000 mg/d iv	Day 10–14, followed by careful tapering	
Plasmapheresis therapy	Day 15, 17, 20, 22, 24	
Levetiracetam		
2 × 500 mg/d	Day 8–9	
2 × 1,500 mg/d	Day 9–17	
2 × 2,000 mg/d	Day 17-ongoing	
Lacosamid 2 × 100 mg/d	Day 9–11	
Midazolam up to 40 mg/h i.v.	Day 10–25	
Phenytoin 3 × 250 mg/d	Day 11–17	
Ketamin up to 300 mg/h i.v.	Day 15–21	
Phenobarbital 2 × 150 mg/d	Day 17–20	
Phenobarbital 2 × 200 mg/d	Day 20-ongoing	
Tiprid 3 × 100 mg/d	Day 24–30	
Dexmedetomidin i.v.	Day 12–34 (recurrent treatment)	
Lorazepam up to 4 × 2.5 mg/d	Day 25-ongoing	
**(C) Imaging**		
CT Scan day 8 and 9:	Normal	
Brain-MRI		
day 8:	– Normal MRI	
day 13:	– Edematous changes in amygdala, hippocampus, and Parahippocampal gyrus predominantly left side	
day 23:	– Progression of edema in amygdala on both sides, hippocampus, and parahippocampal gyrus predominantly left side, additional	
	subcortical FLAIR hyperintensities subinsular bilaterally	
day 38:	– Regression of FLAIR hyperintensities in all areas	
FDG-PET day 35	No signs of metabolically active malignancies. Cerebral hypermetabolism in left amygdala and hippocampal area	
Anti-neuronal antibodies^*^	Negative	Negative
TSH level	10.4 mU/l	0.2–4.3 mU/l
fT3, fT4	Normal	Normal
**(D) Laboratory findings**	**Result**	**Normal value**
Nasopharyngeal swab or tracheobronchial fluid for SARS-CoV-2 RNA Day 8, 10, 16, 22, 29	Negative	Negative
Creatinkinase (μmol/l)	80	45–84
Leukocytecount (10^9^/L)	5.76	3.9–10.2
Thrombocytecount (10^9^/L)	160	150–370
Hemoglobin (g/L)	13.2	12.0–15.4
C-reactive protein (mg/L)	31.9	<5.0
**(E) CSF characteristics**	**Day 7**	**Normal**
CSF cellcount /μl	7	< 5
CSF Lactate (mmol/L)	2.2	1.2–2.1
Glucose (mmol/L)	4.5	2.7- 4.2
Protein (mg/L)	490	< 450
Virus PCRs in CSF	negative PCR for HSV-1/2, VZV, TBEV, Enterovirus	negative
Serologicaltesting	Negative TBEV-IgG and -IgM, antinuclear antibodies, anti-dsDNA antibodies, anti-cardiolipin and β2-glykoprotein antibodies, ANCA	negative
Oligoclonal bands^**^	type 4	type 1
**(F) EEG findings**		
Day 8:	– Generalized slowing, convusive seizure presenting on EEG with rhythmic delta activity	
Day 9:	– Generalized slowing, bifrontal interictal epileptic discharges (IEDs), polytopictriphasic potentials	
Continous EEG day 10–26:	– Periodic discharges with sharp morphology (PD), brief electrographic seizures	
Day 34:	– Polytopictriphasic potentials, no ED, slight regression of pathologies	
Day 36:	– Single ED predominately left side, no change in encephalopathy	
Day 38:	– Single ED predominately left side, no change in encephalopathy	
Day 41:	– Single ED predominately left side, no change in encephalopathy	

* Anti-neuronal autoantibody sera: GAD65, NMDA, GABAAR, GABABR, IgLON5, AMPAR 1/2, DPPX, LGI1, CASPR2, glycin-receptor, mGluR5, and mGluR1; CSF: GAD65, NDMAR, GABAAR, GABABR , IgLON5, AMPAR 1/2, DPPX, LGI1, CASPR2, glycin-receptor, mGLuR5, and mGLuR1; Neural antibody (immunoblot-IgG) sera: amphiphysin, CV2/CRMP5, Ma2/Ta (PNMA2), Ri, Yo, Hu, Recoverin, Sox1, Titin, Zic4, and DNER/Tr; CSF: amphiphysin, CV2/CRMP5, Ma2/Ta (PNMA2), Ri, Yo, Hu, Recoverin, Sox1, Titin, Zic4, and DNER/Tr.

** OCB type 1 = no oligoclonal bands in the CSF and serum, i.e., no intrathecal IgG production; OCB type 2 = oligoclonal IgG bands only in the CSF, i.e., intrathecal IgG production; OCB type 3 = oligoclonal bands in the CSF and serum with additional bands in the CSF, i.e., intrathecal IgG production; OCB type 4 = identical oligoclonal bands in the CSF and serum, i.e., no intrathecal IgG production; OCB type 5 = monoclonal IgG bands in the CSF and serum.

**Figure 2:**
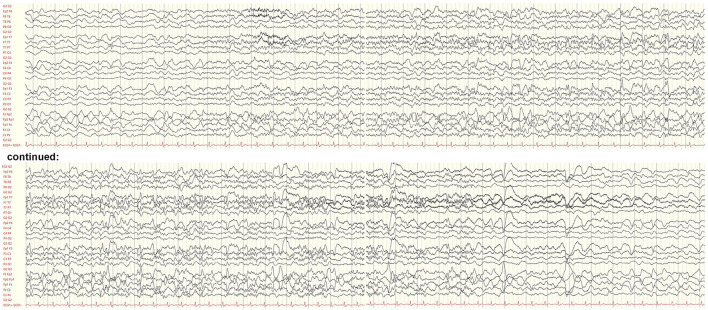
Electroencephalogram on day 8 after symptom onset showing a brief focal seizure. The seizure discharge begins with an evolving fast rhythm with onset in the left temporal leads and interspersed epileptic discharges. There is generalized background slowing following the seizure. Bipolar longitudinal montage, gain 70 μV/cm, base time 3 cm/s, high-pass filter. 53 Hz, low-pass filter 80 Hz, and notch filter 50 Hz.

**Figure 3:**
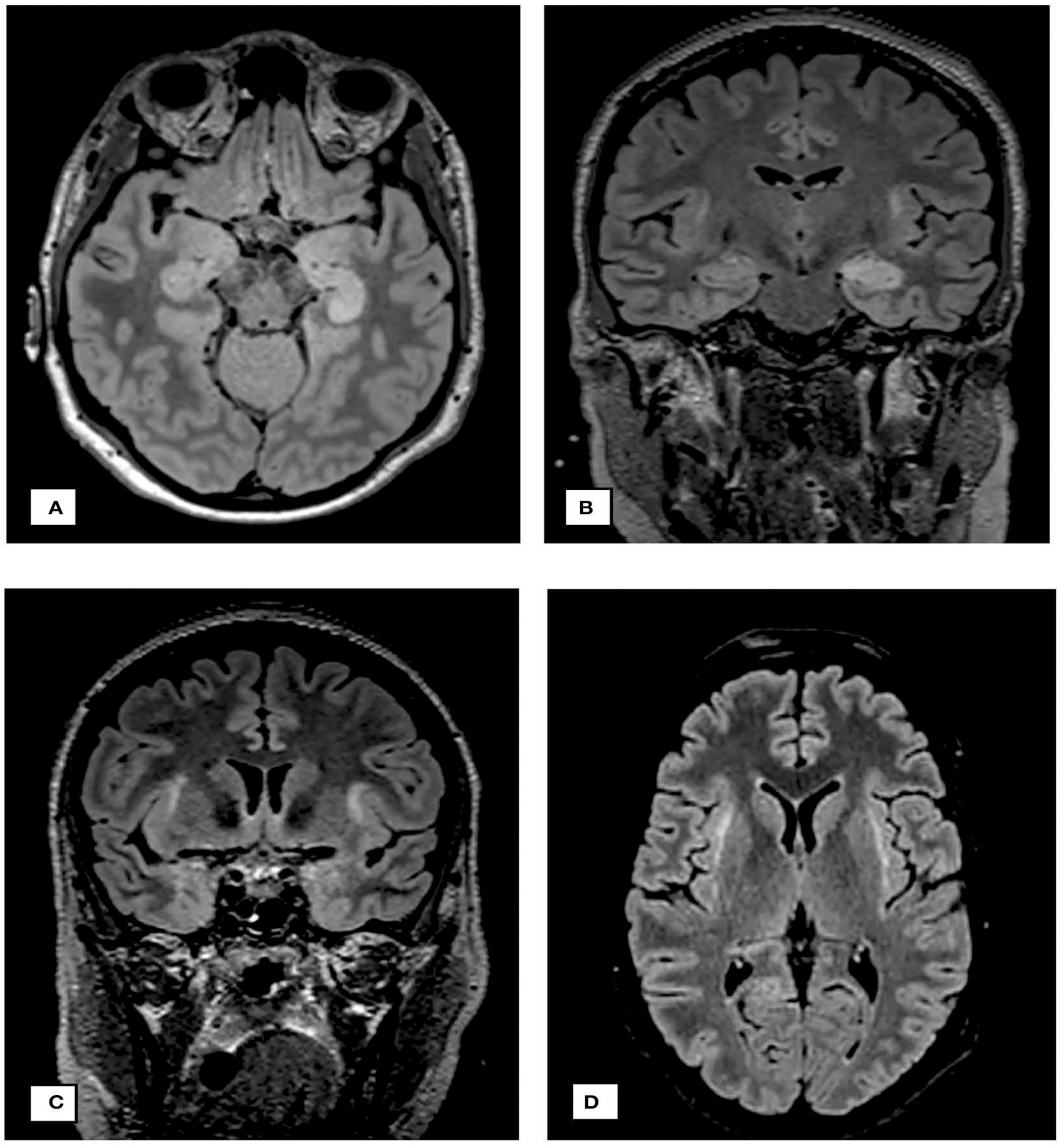
Neuroimaging findings. **(A)** cMRI on day 13: axial FLAIR sequence demonstrating edematous changes in hippocampus and parahippocampal gyrus predominantly on the left side. **(B)** cMRI on day 23: coronary FLAIR sequence demonstrating progression of edema on both sides, the hippocampus and parahippocampal gyrus, additional subcortical FLAIR hyperintensities subinsular on both sides. **(C,D)** demonstrate the claustra hyperintensities.

The electroencephalogram (EEG) initially showed a generalized rhythmic delta activity with superimposed multifocal interictal epileptic discharges mainly over the frontal and right-sided leads. Frequent brief electrographic seizures were also detected (Figure 2). Initial CT and MRI scans were unremarkable. The MRI on day 5 after admission revealed edema in the left mesial temporal lobe, particularly the hippocampus, which became progressive in size 10 days later. Additionally, FLAIR hyperintensive lesions in the bilateral subinsular regions were detected (Figure 3). The FDG PET showed hypermetabolism of the left amygdala and hippocampus and basal pulmonary hypoventilation. Cerebrospinal fluid (CSF) analysis revealed lymphocytic pleocytosis (7 cells/μl), normal protein, glucose, lactate, and total immunoglobulin parameters, and matched oligoclonal bands in the serum and CSF without signs of intrathecal IgG production (Table 1). The blood and CSF screening for common viral and bacterial infections and autoimmune disorders including vasculitis was negative. The diagnostic criteria for a definite autoantibody-negative autoimmune limbic encephalitis according to established criteria were fullfilled (8). However, testing for common anti-neuronal autoantibodies in serum and CSF was negative, including indirect immunofluorescence staining of mouse brain.

Due to recurrent convulsive seizures without regaining consciousness, a diagnosis of new-onset refractory status epilepticus (NORSE) was made. Levetiracetam up to 4 g/d and lacosamide up to 200 mg/d were started. Under continuous EEG monitoring, phenytoin up to 750 mg/d, midazolam up to 0.57 mg/kg/h, and ketamine up to 4 mg/kg/h were added. Phenytoin was later replaced by phenobarbital. Initially, acyclovir 3 × 10 mg/kg body weight/day and ceftriaxone were given until the CSF and serum testing for herpes viral and bacterial CNS infections turned negative. Immunomodulatory treatment with high-dose methylprednisolone (5 days of 1,000 mg/d IV) was started 2 days after hospital admission with subsequent slow tapering over 8 weeks. Because of continued seizures, plasma exchange for over 10 days starting on day 7 after the admission was performed.

Subsequently, the patient's condition rapidly improved beginning on day 12 after the admission; she regained full consciousness and had only infrequent seizures. The follow-up MRI 1 month after the hospital admission showed edema reduction in the hippocampal, amygdala, and external capsule. On discharge, she had persisting moderate mnestic deficits and infrequent seizures (2–3/month). However, after 2 months, the patient was readmitted to our hospital because of deterioration in seizure frequency (2–3 serial seizures) and neuropsychological deficits. She again received high-dose methylprednisolone for 5 days, which led to significant reduction in seizure frequency.

## Discussion and conclusions

We report the first case, to our knowledge, of NORSE due to seronegative autoimmune encephalitis 8 days after vaccination with the mRNA SARS-CoV-2 vaccine. A causal relationship between vaccination and autoimmune encephalitis with NORSE is not established with certainty. Nevertheless, the onset of neurological symptoms 8 days following the vaccination suggests a potential association as well as the hyperintensities of the claustrum as a common finding in NORSE and autoimmune encephalitis (9, 10).

Given the assumed low incidence of autoimmune reactions and their favorable outcome, benefits of vaccination far outweigh the risk of side effects. Importantly, SARS-CoV-2 infection itself may trigger a heterogeneous group of autoimmune encephalitis. Hence, the observation of SARS-CoV-2 vaccine-associated NORSE does not argue against the broad use of SARS-CoV-2 vaccines.

Whether the patient's history of SARS-CoV-2 infection approximately 1 year before the encephalitis may have played a role remains unknown. Our findings should encourage clinicians to consider SARS-CoV-2 vaccination or infection as a potential trigger for autoimmune encephalitis.

## Data availability statement

The raw data supporting the conclusions of this article will be made available by the authors, without undue reservation.

## Ethics statement

Ethical review and approval was not required for the study on human participants in accordance with the local legislation and institutional requirements. The patients/participants provided their written informed consent to participate in this study. Written informed consent was obtained from the individual(s) for the publication of any potentially identifiable images or data included in this article.

## Author contributions

JW collected and interpreted the clinical and radiological data and was a major contributor in the writing and revision of the manuscript. IJ collected and interpreted the clinical and laboratory data and revised the manuscript. GB interpreted the clinical findings and revised the manuscript. MG interpreted the clinical and electroencephalographic findings and revised the manuscript. All authors read and approved the final version of the manuscript.

## Funding

This study was partly supported by the SNF (Grant No: 4078P0_198345, title: Protective and Pathogenic T Cell Immunity During SARS-CoV-2 Infection) and the Loop Zurich, COVID-19 project (Title: SARS-CoV-2-Induced Immune Alterations and their Role in Post-COVID Syndrome).

## Conflict of interest

The authors declare that the research was conducted in the absence of any commercial or financial relationships that could be construed as a potential conflict of interest.

## Publisher's note

All claims expressed in this article are solely those of the authors and do not necessarily represent those of their affiliated organizations, or those of the publisher, the editors and the reviewers. Any product that may be evaluated in this article, or claim that may be made by its manufacturer, is not guaranteed or endorsed by the publisher.

## Abbreviations

COVID-19: coronavirus disease 2019
CSF: cerebrospinal fluid
CNS: central nervous system
EEG: electroencephalogram
ED: epileptic discharge
FDG-PET: 2-fluor-2-desoxy-D-glucose positron emission tomography
FLAIR: fluid-attenuated inversion recovery
HSV-1/2: herpes simplex virus type 1 or 2
IED: interictal epileptic discharge
MRI: magnetic resonance imaging
NORSE: new onset refractory status epilepticus
PCR: polymerase chain reaction
PD: periodic discharge with sharp morphology
RNA: ribonucleic acid
SARS-CoV-2: severe acute respiratory syndrome virus 2
VZV: varicella zoster virus.
